# Mary Elizabeth Hickox Mandels, 90, bioenergy leader

**DOI:** 10.1186/1754-6834-2-22

**Published:** 2009-09-01

**Authors:** Fred Allen, Raymond Andreotti, Douglas E Eveleigh, John Nystrom

**Affiliations:** 1 Science Applications International Corporation, San Diego, CA, USA; 2 116 Freedom Street, Hopedale, MA, USA; 3 Biochemistry & Micxrobiology, SEBS, Rutgers University, New Brunswick, NJ, USA; 4 Millenium Pharmaceuticals, Cambridge, MA, USA

## Abstract

Mary E H Mandels, who spearheaded the US Army's national bioconversion studies for four decades and was an early proponent of conversion of waste biomass to readily bioconvertible sugars for the production of chemicals and transportation fuels such as ethanol, died 17 February 2008 at Natick, MA, USA. She was 90.

## Commentary

**Figure 1:**
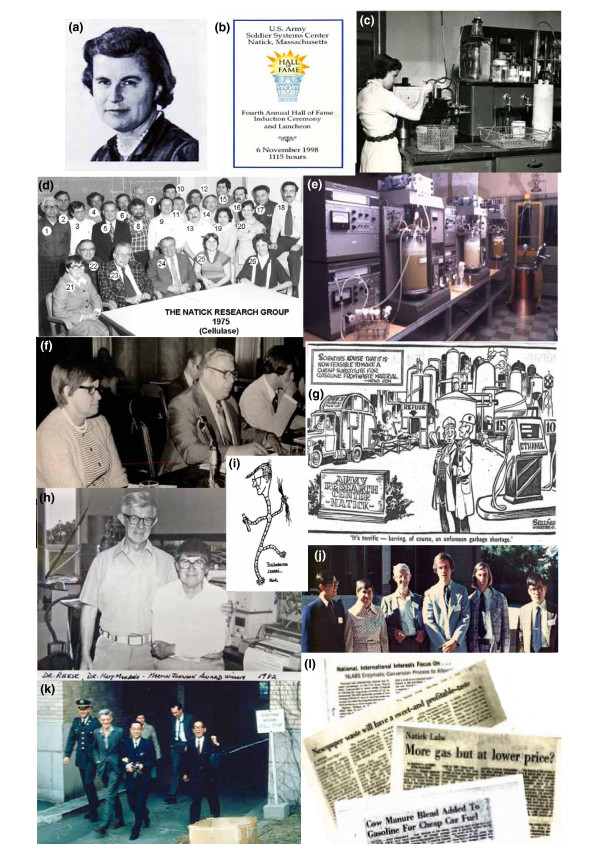
**Images from Mary Mandels' career**. Dr Mary Mandels was one of Natick's original employees of the Pioneering Research Laboratory. Her research included screening fungal cultures for their ability to produce cellulase and related enzymes. The purpose of the research was to increase the understanding of the deterioration of materials caused by fungal attack. From left to right: **(a) **Mary Mandels, 1917 to 2008: **(b) **US Army Hall of Fame, 1988; Natick Hall of Fame program cover; **(c) **Mary Mandels at the bench, Pioneering Research Laboratory, Natick, 1957; **(d) **The US Army Natick Pioneering Research Group, 1975: 1. Ed Black, 2. Dick Erickson, 3. David Sternberg, 4. Fred Allen, 5. Frank Snyder, 6. Frank Bisset, 7. Charlie Macy, 8. Ray Andreotti, 9. Curt Blodgett, 10. Marty Foncello, 11. Dr John Walsh, 12. Dr Aaron Bluhm, 13. military researcher, 14. Ben Gallo, 15. John Medieros, 16. Charles Roche, 17. Dr Carmine DiPetro, 18. Tom Tassinari, 19. Rosa Linda Bagalawis, 20. military researcher, 21. Mary Mandels, 22. Bob Mortenson, 23. Phil Hall, 24. Leo Spano, 25. Beverly Grant, 26. Edie Blodgett; **(e) **the Cellulase Laboratory, Pioneering Research Laboratory, 1975 (overview of the fermentor laboratory: six 14 litre fermentors (New Brunswick Scientific Co., Edison NJ, USA) plus a 30 litre seed and two production fermentors of 250 litre and 400 litre capacity, respectively); **(f) **US Congressional Energy Hearings, 1974: Congressman William Proxmire with Mary Mandels, Leo Spano and John Nystrom testifying (Congressman Proxmire was famed for his Golden Fleece award to those agencies that he thought had financially 'fleeced' the Government with arcane science; the US Army Natick program passed the 'fleece test' with flying colours); **(g) **The Natick Recycling Program as viewed by the local Press, 1974 (courtesy Worcester Telegram and Gazette); **(h) **Elwyn Reese and Mary Mandels: laboratory photo on the occasion of their receiving the Marvin Johnson Award, American Chemical Society, 1982; **(i) **insert cartoon of Elwyn Reese depicted as the mould *Trichoderma *and entitled 'The Fungus Factory' (Ray Andreotti artist); **(j) **Natick Cellulase Researchers: 'Cellulose as a Chemical & Energy Resource', University of California - Berkeley, 1974 (Andy Huang (postdoctorate), Mary Mandels, Elwyn Reese, John Nystrom, Bob Andren, Chul Kim (Korean AEC); **(k) **Natick Cellulase Group with visitors at Massachusetts Institute of Technology, May 1962: front row: (left to right) Chico Bomschell, Elwyn Reese, K Nisizawa (Tokyo, Japan), Nobuo Toyama (Miyasaki, Japan); second row: Mary Mandels, Keith Selby (Birmingham, UK); visitors while attending Advances in Enzyme Hydrolysis of Cellulose and Related Materials (American Chemical Society and US Army Symposium in Washington, DC); **(l) **the range of interests the US Army research programs: a selection of newspaper headings. Images courtesy of (a) Mandels' family; (b-e) US Army Pioneering Research Laboratory; (f) J Nystrom; (g) Worcester Telegram and Gazette; (h-j, l) Alfred A Allen; (k) D Eveleigh.

Dr Mandels spent her lifelong research career at the US Army Natick Research Laboratory (NLABS), just west of Boston, MA, USA (Figure 1a, b, c). Initially assigned to assess microbial deterioration of Army materials, her studies crisscrossed fundamental study of the cellulase enzyme from diverse microbial strains, to enzyme structure, to synergism between hydrolase components, and to large-scale enzyme production. Underpinning her studies was the need for a facile yet meaningful cellulase assay that took into consideration the insoluble and variable degree of crystallinity of the substrate, its changing nature during assay, and additionally the synergistic interaction of the multiple enzymes during hydrolysis. With her filter paper assay she mastered this broad requirement early on, as recognised in *Citation Classics *[1] (330 citations make it this journal's most cited paper in 1988).

Trained in botany (BS, 1939) and plant physiology [2] with minors in biochemistry and microbiology at Cornell University, NY, USA, Dr Mandels' background complemented the array of projects in the Pioneering Research Laboratories at NLABS. The cellulase enzyme complex was of keen military interest, it being a prime cause of the microbial deterioration of fibre and fabric: tents, cordage and clothing. She helped clarify the multicomponent nature of the cellulase complex. In the Food Science Laboratory she addressed the production of unconventional foods such as microbial single cell protein (SCP) and laboratory plant cell vegetable culture in consideration of military operation in hostile environments (for example, Antarctica), and then with the world energy crunch of the mid 1970s developed the US Army Natick recycling program centred around conversion of waste cellulosics to sugar to be fermented to ethanol, an alternative transportation fuel (gasohol = 10% ethanol) or other energy-related chemicals.

Mary, as she was known by colleagues and friends, was the eldest of five children, born to Sherman Gray and Mary Bolger Hickox at Middletown Springs, VT, USA on 12 September 1917. The family soon moved to Waterbury, CT, USA. Growing up in the Great Depression, Mary was a leader to her three sisters Corinne, Alice and Eleanor and brother Sherman, and they remained close throughout their lives. Her father was appreciative of her educational goals, yet though she had won acceptance to Cornell he still had to be nudged by Mary's science teacher to give her the support to attend college. Subsequently her mother died while she was a freshman, and then he insisted that Mary continue with her studies rather than help at home with the raising of her siblings. In 1942 she married fellow Cornell graduate student Gabriel Mandels, her studies (1939 to 1947) being disrupted by World War II. After the war and with their family established, Mary began her lifelong research career at the US Army Natick Laboratories in 1955. Her husband Gabriel had previously worked at what was then the Quartermaster Research Laboratories in Philadelphia.

The Mary Mandels' cellulase saga began as a result of the massive rotting of military equipment during the South Pacific campaign of World War II. The Army established a program to understand its cause and means to control it. That program, initially at the Quartermaster General Laboratories in Philadelphia, PA, USA, was transferred to the new Quartermaster Research and Development Command at Natick in 1954. Mary Mandels began her career joining Elwyn Reese soon after in 1955. By that time, of the 14,000 moulds in the Quartermaster Fungal Collection *Trichoderma *sp. QM6a (later *T. reesei *in honour of Elwyn Reese [3] (Figure 1i).) was noteworthy in producing a secreted cellulase with the outstanding quality to degrade native crystalline cellulose. Mary's role was a fundamental study in helping clarify the multicomponent nature of the cellulase enzyme complex. The synergistic action between the exosplitting and endosplitting components became evident, though arguments ensued as to the degree of synergism in that their perfect purification was somewhat dubious due to the lack of resolution by starch block electrophoresis used in that era. Only later with the subsequent cloning of the individual genes were such issues finally resolved. Induction of an enzyme in order to attack an insoluble substrate was an intriguing concept, made all the more so when in the middle of their research program *T. reesei *stopped producing cellulase. With the help of Fred Parrish, the cause was tracked to a change in the manufacture of glucose from starch, the hydrolysis changing from use of acid to use of amylases. Acid hydrolysis produced reversion products including the β-1,2-glucose dimer, sophorose, which turned out to be a highly active cellulase inducer of *T. reesei*. Thus initially commercial 'glucose', which happened to contain 0.006% sophorose, was apparently an inducer of cellulase. The new enzymatically-derived and more refined glucose could not be used in media for cellulase production [4]. The finding that sophorose was an inducer was key in subsequent studies, while tracking its presence in old commercial glucose samples is a now classic scientific 'detective' story. Dr Mandels also addressed means to inhibit cellulases, uncovering a series of natural plant inhibitors that later were shown to be of significance in the regulation of plant growth.

Transferred to the Food Science Laboratory (1962), Dr Mandels' role was to address production of foods for the military in 'hostile' environments, the latter ranging from Antarctica to the battlefield to extended space missions. The preparation of 'in house' unconventional foods was addressed through consideration of plant culture (beans, lettuce and carrots) and single cell protein (SCP) in fermentors, both exciting topics at that time. The latter included consideration of cellulolytic microbes.

Mary was reassigned to the Bioengineering, Science and Advanced Technology Laboratory in 1971, and with Elwyn Reese retiring in 1972 she went on to head this group (Figure 1d). With the onset of the oil embargo of the 1970s came the search for further energy resources. Mary spearheaded the US Army Natick Program for the enzymatic conversion of agricultural and wood wastes to sugar, to be fermented to chemicals including ethanol, to be used as alternate liquid transportation fuels. Thus developed the renowned Natick Bioenergy program under her aegis (Figure 1j). The program was all inclusive. Enhanced production of cellulase was achieved by the development of hypercellulase-producing mutants, optimisation of culture media through use of surfactants and inducers, and enhanced production was further gained by use of fed-batch and two stage continuous fermentation that included scale-up to 400 litre fermentors and 250 litre hydrolysis reactors (Figure 1e). Enzyme reuse was addressed. Numerous municipal and agricultural wastes were pretreated and evaluated with regard to their susceptibility to hydrolysis. The optimised cellulases produced up to 10% glucose solutions and attempts were made to couple such production to yeast fermentation.

**Figure 2:**
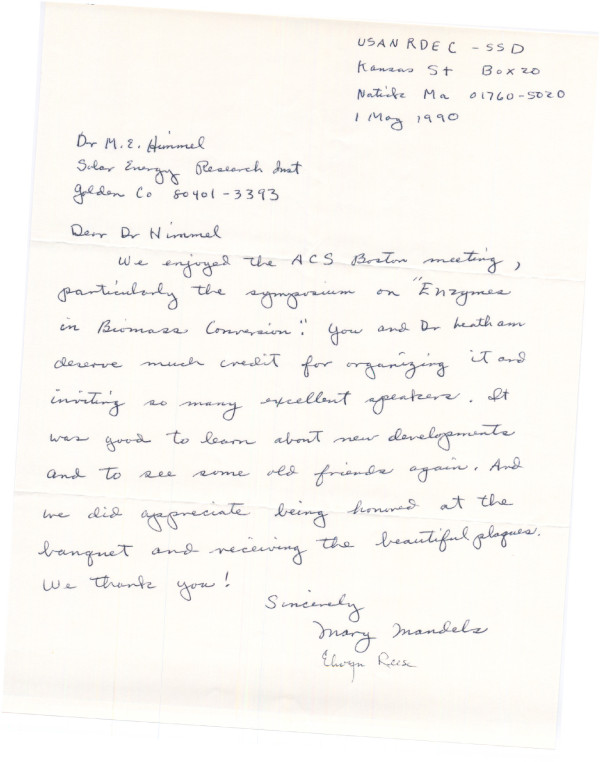
**Letter of appreciation, dated 1 May 1990, written by Mary Mandels to Mike Himmel for organising a special symposium on 'Enzymes in Biomass Conversion' at the 1990 American Chemical Society meeting in Boston, MA, USA**. The letter is signed by both Mary and Elwyn Reese. Courtesy of Michael E Himmel, National Renewable Energy Laboratory, Golden CO, USA.

**Figure 3:**
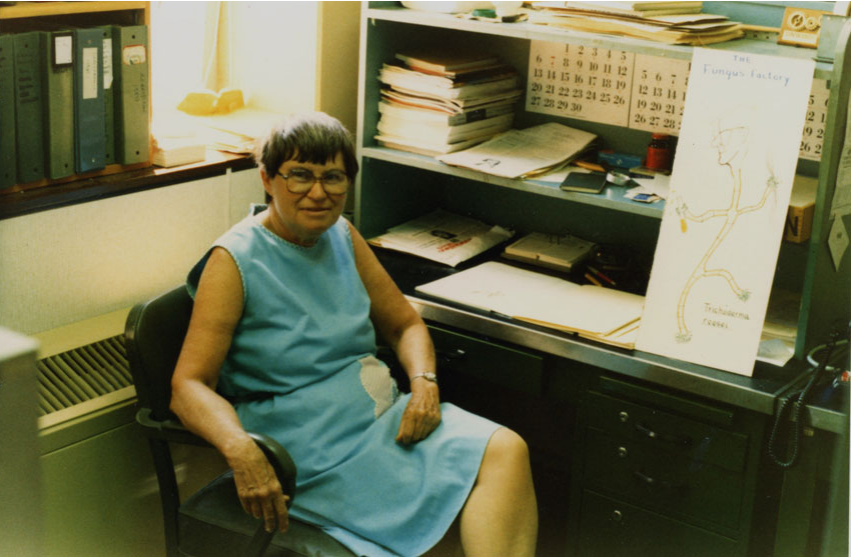
**Mary Mandels, 1981**. Head of Bioengineering, Science & Advanced Technology Laboratory, US Army, Natick, MA, USA. Courtesy of Ray Andreotii.

The importance of the 'wastes to transportation fuel' approach was widely recognised by the press, bringing national fame to the US Army Natick program (Figure 1g, l). Mary, with Leo Spano and John Nystrom, were key advisors at the US Congressional Hearings in 1974 on alternate energy resources. The Natick Program was well received, and did not receive the classic but derogatory William Proxmire 'Golden Fleece' Award for waste of government money (Figure 1f). The Natick Laboratory had become one of the world's cellulase centres [8,9]. Such international fame attracted a series of international workers to Natick for training and discussion (Figure 1k). In like manner, Mary's expertise was widely sought and she lectured and presented short courses in China, Finland, Guatemala, India, Japan, South Africa and Mexico. Though officially retiring in 1984 she remained active in the laboratory until 1994 (Figures 2 & 3). Mary was a leader of national bioenergy studies.

She is survived by her children, Joan Mandels Roche (and John Roche), Suffield, CT, USA, and Peter Mandels, Sherborne, MA, USA, her granddaughter, Susan Roche, Portland, ME, USA, grandson Stephen Roche, Spring Valley, CA, USA, and her sisters Eleanor Hickox, Isle of Wight, UK and Alice Selzer, Oxnard, CA, USA. Her husband, Gabe, predeceased her in 1981.

Mary and her family were keen hikers, skiers and campers. She was an enthusiastic gardener and perceptive naturalist. To stop by at her Natick home was always refreshing, often enhanced by a walk with her at the Broadmoor Audubon Sanctuary, Natick or the Garden in the Woods, Framingham where she bubbled forth with her insight of the local birds, plants and mushrooms. Mary was respected as a naturalist and it gave her great pleasure to have had an insect named in her honour: *Neoloboptera mandelsae *[5] (*Neoloboptera mandelsae *should not be confused with *Salganea mandelsi*, named in honour of her husband Gabriel R Mandels [6]). Roth commented that 'the species is dedicated to Mary Mandels, microbiologist, pioneer in the study of cellulase, and dear friend'.

Mary's breadth of knowledge was remarkable, and she was *au courant *and quite outspoken on current and Army affairs. In the latter vein, Mary was concerned with the interactive role of civilians working for the military and expressed her experiences through *The history of the pioneering research laboratory *(unpublished) and *Reflections on the United States military 1941-1987 *[7]. The first is a superb 'must read' for any scientist working at the Natick Laboratories to gain a historical perspective; factual and with occasional pithy comment. The second is a heartfelt record of concerns that she thought should be brought to focus in order to enhance future civilian/Army joint ventures. The Army refused to publish either of them (they will be made available through a Rutgers Biochemistry & Microbiology website . The latter article is reproduced in this issue (7). Her granddaughter's memoire will also be available on this site. (Susan Roche: *Dr Mary Hickox Mandels, a pioneer in microbiology, with a passion for life*, unpublished).

There are legendary stories from the Natick program. The 'waste' substrates included both old shredded money and hydropulped confidential government documents (several tons per day). In like manner, the front pages of 50 of the top newspapers were processed (ball milled and enzymatically hydrolysed to glucose) to sugar, the comment being that the Army made 'the front page news totally digestible'. Other legends need to be put to rest. Thus reporters wanted to say that the original mould, *Trichoderma *sp., was isolated from a dead rotting soldier whereas it was in fact from a rotting cotton shelter half (bivouac) from Bougainville Island in the Solomon Islands [8].

Mary clearly broke the invisible ceiling promotion barrier that thwarted so many women of her era. For her insight and accomplishments Dr Mandels received continual acclaim: the Civil Servant of the Year (Northeast) 1962, Gold Star for Research 1962 and 1972, US Army Research and Development Achievement Award 1972, Federal Employee of the Year 1973, Exceptional Civilian Service Award 1977, the Marvin Johnson Award, Fermentation Division, American Chemical Society with Elwyn Reese 1982 (Figure 1h), and the US Army Natick Research, Development and Engineering Center Federal Women's Program Award 1991. Peer recognition of her cellulase studies was clear through acclaim of her studies in *Citation Classics *[1]. After retiring in 1984, her fame lived on and Mary was inducted into the US Army Hall of Fame (Natick) 1998. Mary Mandels was vibrant and passionate, sensitive, supportive and encouraging, besides a most accomplished scientist. She was truly the matriarch of cellulase.
